# Human Pluripotent Stem Cells: Applications and Challenges in Neurological Diseases

**DOI:** 10.3389/fphys.2012.00267

**Published:** 2012-07-20

**Authors:** Youssef Hibaoui, Anis Feki

**Affiliations:** ^1^Stem Cell Research Laboratory, Department of Obstetrics and Gynecology, Geneva University HospitalsGeneva, Switzerland; ^2^Service De Gynécologie Obstétrique, HFR Fribourg – Hôpital CantonalFribourg, Switzerland

**Keywords:** pluripotent stem cells, neurological diseases, neurodegenerative diseases, neurodevelopmental diseases, disease modeling, drug screening, regenerative medicine

## Abstract

The ability to generate human pluripotent stem cells (hPSCs) holds great promise for the understanding and the treatment of human neurological diseases in modern medicine. The hPSCs are considered for their *in vitro* use as research tools to provide relevant cellular model for human diseases, drug discovery, and toxicity assays and for their *in vivo* use in regenerative medicine applications. In this review, we highlight recent progress, promises, and challenges of hPSC applications in human neurological disease modeling and therapies.

## Introduction

A major challenge in human neurological diseases is the understanding of the detailed mechanisms responsible for the clinical features. In fact, the lack of access to the affected tissue has limited the study of the molecular and cell biological aspects of the pathogenesis. For instance, several studies reported genotype-phenotype correlations using genetic analysis approaches; however, in most of the studies the molecular mechanisms responsible for the pathogenesis were not fully addressed. Human cell lines and tissues have been used for the study of the pathogenesis of such diseases; however these models are often not relevant as they usually do not recapitulate the human phenotype. Indeed, “healthy” fibroblasts from patients affected by neurological diseases are readily available but these cells are not the neural cells of interest. Neural stem cells (NSCs) have been isolated from human fetal and adult brains in post mortem conditions. While these cells might be an excellent model for the study of human neural development in physiological and pathological conditions (Svendsen et al., 1998; Carpenter et al., 1999; Vescovi et al., 1999; Bahn et al., 2002), they are scarce and do not support systematic analysis. Moreover, long term culture of NSCs has been shown to promote glial differentiation pattern at the expense of neuronal differentiation (Anderson et al., 2007) and to promote cell senescence (Bhattacharyya et al., 2009). Therefore, such effects reduce the potential of these cells for research and therapy. Many notable insights into the neurological disorders have been provided via studies using animal models (mouse principally; Gama Sosa et al., 2012). For some of them, animal models display the neurological phenotype (behavioral abnormalities, anatomical, and cellular perturbations) consistent with human disease (Baker, 2011; Winner et al., 2011). However, the others are not accurately recapitulated in animal models and thus cannot be investigated by this approach (Schnabel, 2008; Scott et al., 2008; Schulz et al., 2009; Chesselet and Richter, 2011). In fact, several neurological phenotypes such as mental retardation or cognitive behavior have human specific manifestations. The incomplete synteny between animal and human genetics together with behavioral and physiological discrepancies account for this.

An innovative way to study human neurological diseases is through the use of human pluripotent stem cells (hPSCs; Park et al., 2008; Mattis and Svendsen, 2011; Zhu et al., 2011). These cells are defined by two criteria: (i) their ability to continually self-renew and (ii) their ability to differentiate into cells of the three primitive germ layers (endoderm, mesoderm, ectoderm). These cells include embryonic stem cells (ESCs), induced pluripotent stem cells (iPSCs), embryonic germ cells, and embryonic carcinoma cells. In fact, the generation of disease-specific hPSCs offers the opportunity to reproduce normal and pathological neural tissue development (Lee and Studer, 2010). The differentiation of hPSCs into multiple neuronal lineages is a powerful tool for studying early embryonic neurogenesis and the mechanisms involved in the pathogenesis of human neurological diseases. Also, it provides a unique opportunity to generate a number of cells of neural lineage for regenerative medicine (Lee and Studer, 2010; Lee et al., 2010) and should provide new therapies for such diseases.

In this review, we explore the growing interest in using hPSCs and in particular human ESCs (hESC) and iPSCs: *in vitro* as research tools for modeling human neurological diseases and drug screening and *in vivo* in regenerative medicine. We will also highlight the challenges and limitations in the field.

## Human Embryonic Stem Cells

**Figure 1:**
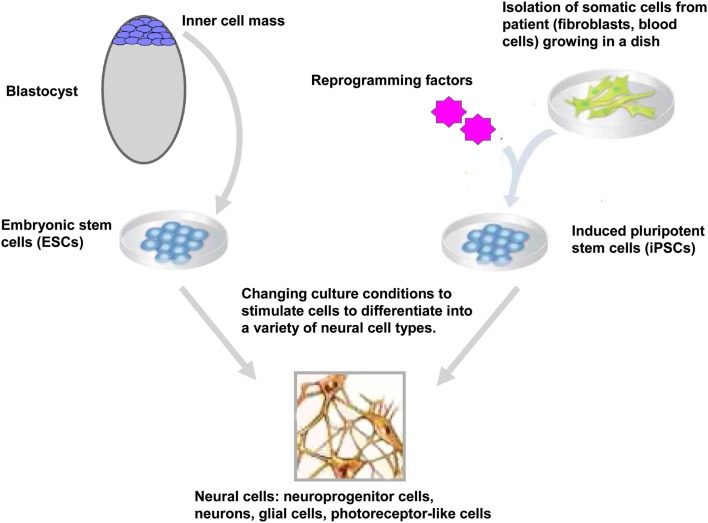
**Generation and neural differentiation potential of pluripotent stem cells**. Human embryonic stem cells (hESCs) are derived from the inner-cell mass of blastocyst stage embryos. Human induced pluripotent stem cells (hiPSCs) are reprogrammed from somatic cells after the ectopic expression of reprogramming factors. After neural induction using specific stimuli, hESCs, and hiPSCs differentiate into neuroprogenitor cells and further mature into neurons, glial cells, retinal pigment epithelium, and other neural cells (only cells of the neural lineage are represented).

Embryonic stem cells are derived from the inner-cell mass of blastocyst stage embryos (Figure 1). Historically, since the isolation of the first mouse embryonic stem cells (mESC) in 1981 (Evans and Kaufman, 1981), it took another 17 years before the generation of the first hESC lines (Thomson et al., 1998). ESCs held great promise in biology and medicine as these cells showed the potential to proliferate over prolonged period of time and to differentiate *in vivo* and *in vitro* into derivatives of the three germ layers endoderm, ectoderm, and mesoderm (Keller, 2005; Murry and Keller, 2008). Typically, ESCs are maintained in the undifferentiated state by co-culture on fibroblasts cells (also called feeder cells) where they retain their ability to self-renew indefinitely. When these ESCs are removed from the feeder cells and transferred in suspension condition, they aggregated to form embryoid bodies (EBs) that contain derivatives of the three germ layers. In this regard, huge efforts have been made to simplify the protocol for maintaining the ESCs in the undifferentiated state; such as culture of ESCs on Matrigel™ in the absence of feeder cells (Xu et al., 2001) or the addition of a selective inhibitor of Rho-associated coiled-coil kinase (p160-ROCK) to the culture medium after dissociation and passaging of the ESCs (Watanabe et al., 2007). At least three general approaches have been used to promote neural differentiation of ESCs: as EBs, as adherent cells and in co-culture with appropriate support cells or in a combination of these three approaches (Reubinoff et al., 2001; Tabar et al., 2005; Lee et al., 2007a). More recently, a feeder-free monolayer culture method for neural differentiation has been established via dual inhibition of SMAD signaling. This approach uses a combination of bone morphogenetic protein 4 inhibitors (such as Noggin or Dorsomorphin) and inhibitors of Lefty/activin/TGFβ pathway (such as SB431542) to improve the efficiency of the differentiation (Chambers et al., 2009). At present, differentiation protocols do not exist for the generation of all cell types of the central nervous system (CNS), however over the past decade progress has been made for directed differentiation of hESCs into several neural cell types of the CNS (Suter and Krause, 2008; Liu and Zhang, 2011; see also in the same issue Martinez et al., 2012) including specific subtypes of neurons (Wichterle et al., 2002; Ying et al., 2003; Yan et al., 2005; Lee et al., 2010), oligodendrocytes (Hu and Zhang, 2009, 2010; Hu et al., 2009), astrocytes (Krencik et al., 2011; Liu and Zhang, 2011), and retinal cells (Meyer et al., 2009, 2011; Osakada et al., 2009; Lamba and Reh, 2011).

## Reprogramming of Somatic Cells into a Pluripotent State

Epigenetic reprogramming of somatic cells into a pluripotent state has been achieved using several approaches including nuclear transplantation, cell fusion (for review see Jaenisch and Young, 2008; Yamanaka and Blau, 2010) and more recently, direct reprogramming by the expression of reprogramming factors. Takahashi and Yamanaka reported a significant advance in the stem cell field with the reprogramming of somatic cells into ESC-like cells (Figure 1). They demonstrated that the ectopic expression of four factors *Oct4*, *Sox2*, *klf4*, and *c-Myc* reprogrammed mouse embryonic fibroblasts into iPSCs (Takahashi and Yamanaka, 2006). As ESCs, these iPSCs could differentiated *in vivo* and *in vitro* into cells of the three germ layers and generate chimeras when injected into blastocyst embryos (Takahashi and Yamanaka, 2006). One year later, two independent groups had successfully reprogrammed human fibroblasts into human iPSCs (hiPSCs) using two different sets of reprogramming factors; the former using *Oct4*, *Sox2*, *klf4*, and *c-Myc* (Takahashi et al., 2007) and the latter using *Oct4*, *Sox2*, *Nanog*, and *Lin 28* as reprogramming factors (Yu et al., 2007). Direct reprogramming is a slow and inefficient process with efficiencies ranging from 0.002 to 0.02% (Takahashi et al., 2007; Yu et al., 2007). During and after this stochastic process (Hanna et al., 2009), the generated iPSCs have to be carefully tested for their pluripotency properties and their differentiation potentials. In particular, the ESC-specific transcription factors *Oct4* and *Nanog* have to be demethylated upon reprogramming of the somatic cells into iPSCs (Takahashi et al., 2007; Mikkelsen et al., 2008; Ebert et al., 2009). The differentiation into derivatives of the three germ layers *in vitro* and *in vivo* (in the teratoma formation assay) is also a necessary hallmark of a fully reprogrammed iPSCs. Moreover, the efficiencies of iPSC generation and differentiation depends on the stoichiometry of the reprogramming factors (Papapetrou et al., 2009; Tiemann et al., 2011) and the silencing of the vector-encoded reprogramming factors (Maherali and Hochedlinger, 2008; Ramos-Mejia et al., 2010).

As the introduction of the reprogramming factors using lentivirus or retrovirus for the generation of iPSCs may render these cells unuseful for research applications and regenerative medicine due to potential insertional mutagenesis, non-integrating reprogramming strategies have been developed including plasmids (Okita et al., 2007), episomal vectors (Yu et al., 2009), piggyBac transposition (Woltjen et al., 2009), Cre- or Flp-recombinase-based excisable viruses (Soldner et al., 2009; Voelkel et al., 2010), membrane soluble protein-induced methods (Kim et al., 2009; Zhou et al., 2009), modified RNA (Warren et al., 2010), and miRNA (Anokye-Danso et al., 2011). Reprogramming into iPSCs has been also achieved using small molecules that can either replace reprogramming factors or enhance reprogramming efficiency (Feng et al., 2009). Up to now, iPSCs have been reprogrammed from several types of somatic cells including fibroblasts (Takahashi et al., 2007; Park et al., 2008; Ebert et al., 2009), neural progenitor cells (Shi et al., 2008), keratinocytes (Aasen et al., 2008), peripheral blood (Loh et al., 2009), pancreatic B cells (Stadtfeld et al., 2008), and hepatocytes (Aoi et al., 2008). Like hESCs, hiPSCs have been successfully differentiated into NPCs (Chambers et al., 2009; Liu and Zhang, 2011), specific subtypes of neurons (Di Giorgio et al., 2008; Dimos et al., 2008; Ebert et al., 2009; Soldner et al., 2009), oligodendrocytes (Czepiel et al., 2011), astrocytes (Krencik et al., 2011), and retinal cells (Buchholz et al., 2009; Meyer et al., 2009, 2011; Osakada et al., 2009; Jin et al., 2011).

## Applications

**Figure 2:**
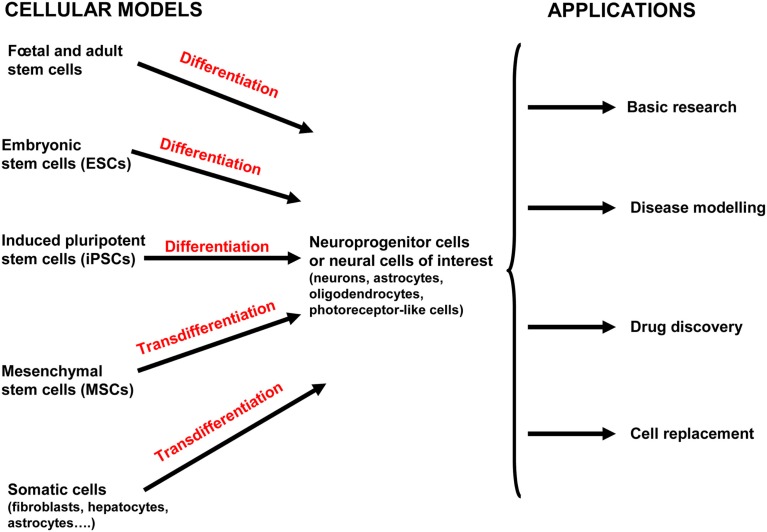
**Current and potential approaches used for human neurological disease study and therapy**. Both human embryonic stem cells (hESCs) and induced pluripotent stem cells (hiPSCs) can differentiate into neuroprogenitor cells and/or further mature into the neural cells of interest (neurons, oligodendrocytes, astrocytes, and photoreceptor-like cells). Mesenchymal stem cells (MSCs) have been isolated from various tissue including placenta, adipose tissue, lung, bone marrow, blood, and the umbilical cord (and possibly others). MSCs can be directly converted into cells of the ectodermal lineage by transdifferentiation (also called plasticity). Neural stem cells (NSCs) are isolated from fetal and adult brains of aborted fetuses and adult brains in post mortem conditions. Adult NSCs are mostly obtained from two regions of the adult brain where neurogenesis occurs: the subventricular zone of the lateral ventricle and subgranular zone of the dentate gyrus in the hippocampus. Induced neural cells (iN cells) are generated from the transdifferentiation of somatic cells from the same lineage or another one without the prior reprogramming into pluripotent cells. All these cells provide valuable model for basic developmental research, modeling diseases, high-throughput drug screening, and cell-based therapies.

In this section, we discuss four major applications of hPSCs that will advance our understanding of human neurological diseases through deciphering the targets and mechanisms involved in the pathogenesis. The first two applications are the study of neural development and differentiation processes in physiological and pathological contexts. Then, the identification of the detailed mechanisms that contribute to the pathogenesis of the disease will provide targets for drug screening and cell-based therapies for neurological diseases (Figure 2).

### Basic developmental biology

Over the last decade, hPSCs have emerged as a valuable and powerful material for studying the pathways governing human embryogenesis and development (Keller, 2005). Such studies were previously unattainable due to technical and ethical concerns regarding the use of human fetuses. Thus, hPSCs enable the investigation of the basic mechanisms involved in pluripotency, neural fate specification, and differentiation (Munoz-Sanjuan and Brivanlou, 2002; Levine and Brivanlou, 2007; Hanna et al., 2010). The knowledge accumulated by embryologists from frog, fish, chicken, and mouse embryos has allowed the development of strategies to direct neural fate specification from hPSCs *in vitro* (Munoz-Sanjuan and Brivanlou, 2002; Stern, 2005, 2006; Levine and Brivanlou, 2007). As in the developing embryo, neural differentiation of hPSCs appears to be a default lineage differentiation when self-renewal is not maintained. It explains why early protocols used spontaneous differentiation for generating neural cells even with low efficiency (Reubinoff et al., 2001; Tropepe et al., 2001; Stern, 2006). Subsequent studies have used factors and patterning signals that mimic embryogenic neurogenesis to improve fate specification and differentiation efficiencies. Bone morphogenetic proteins (BMPs), wingless-type MMTV integration site family (WnT), and Smad signaling are pathways that suppress the induction of ectoderm (Munoz-Sanjuan and Brivanlou, 2002). Based on this, addition of BMP, WnT, or Smad inhibitors promote specification of hESCs into neuroectoderm (Pera et al., 2004; Watanabe et al., 2005; Smith et al., 2008). This conversion was further improved by dual inhibition of SMAD signaling using noggin and SB431542 (Chambers et al., 2009). At the same time, elegant work has demonstrated the successful differentiation of hPSCs into specific subtypes of neurons (Ying et al., 2003; Di Giorgio et al., 2008; Dimos et al., 2008; Chambers et al., 2009; Ebert et al., 2009; Soldner et al., 2009; Lee et al., 2010; Liu and Zhang, 2011), oligodendrocytes (Hu and Zhang, 2009, 2010; Hu et al., 2009), and astrocytes (Krencik et al., 2011; Liu and Zhang, 2011). Thus, excitatory projection neurons (Watanabe et al., 2005; Eiraku et al., 2008; Gaspard et al., 2008) and cortical interneuron progenitors (Maroof et al., 2010) have been generated from ESCs using brain development principle. When transplanted into postnatal cortex, these cortical interneuron progenitors migrated, integrated into the local circuitry and displayed morphological and electrophysiological properties of mature interneurons (Maroof et al., 2010). Therefore, hPSCs provide an unprecedented opportunity for basic research focused on neuronal activity, migration, dendritogenesis, synaptogenesis, and integration to circuitry *in vitro* or when transplanted *in vivo* (Maroof et al., 2010; Brennand et al., 2011; Kim et al., 2011a). Further understanding of the signaling pathways governing these processes in hPSCs will provide new insights into human neurodevelopment and the functional integration of transplanted cells.

### Modeling human neurological diseases

Human neurological diseases can be modeled using hESCs, essentially by two approaches. The first approach is by inducing a mutation in healthy hESCs. Perhaps the best example for this is the generation of hESCs for the modeling of Lesch–Nyhan syndrome, a disease caused by a mutation in the *HPRT1* (*hypoxanthine-guanine phosphoribosyltransferase*) gene that triggers an overproduction of uric acid, causing gout-like symptoms, and urinary stones, in addition to neurological disorders. Urbach et al. (2004) succeeded in generating a hESC-based model that recapitulates in some extent the characteristics of Lesch–Nyhan disease, by mutating the *HPRT1* gene in hESCs using homologous recombination. The second approach is through the identification of hESCs derived from embryos affected by genetic disorders during pre-implantation genetic diagnosis (PGD; Ben-Yosef et al., 2008; Stephenson et al., 2009). In this regard, hESC lines has been derived after PGD for a broad range of neurological diseases including Fragile X syndrome (FXS; Verlinsky et al., 2005; Eiges et al., 2007; Frumkin et al., 2010; Tropel et al., 2010), Spinocerebellar ataxia 2 (Tropel et al., 2010), Huntington’s disease (HD; Verlinsky et al., 2005; Mateizel et al., 2006; Tropel et al., 2010), Down syndrome (DS; Biancotti et al., 2010; Sharon et al., 2011), Gaucher’s syndrome (Frumkin et al., 2010), Charcot Marie Tooth disease (Mateizel et al., 2006), X-linked adrenoleukodystrophy (X-ALD; Verlinsky et al., 2005), familial amyotrophic lateral sclerosis (ALS), neurofibromatosis type 1, Patau syndrome (Biancotti et al., 2010), and possibly others (Table 1).

**Table 1:** **Neurological diseases in which hPSCs (either hESCs or hiPSCs) have been derived from embryos or patients**.

Disease	hPSC model used	Molecular defects associated with the disease	Phenotype reported	Reference
Angelman’s syndrome (AS)	iPSC	15q11–13	*UBE3A* genomic imprinting in AS-iPSC-derived neurons	Chamberlain et al. (2010)
Alzheimer’s disease (AD)	iPSC	Unknown or mutation/duplication in *APP*, *PS1*, *PS2*	High levels of amyloid-β(1-40), phospho-tau (Thr231), and active glycogen synthase kinase-3β (aGSK-3β) in AD-iPSC-derived neurons	Yagi et al. (2011), Israel et al. (2012)
Charcot Marie Tooth (CMT)	ESC	*CMT1*, *PMP22*, *GJB1*, *MPZ*, *MFN2*, *GJB1*, *GDAP1*, *NDRG1*, *HK1*, *SH3TC2*, *GDAP1*, *GJB1*, and *MPZ* (depending on the type of CMT)	Not determined	Mateizel et al. (2006)
Down syndrome (DS)	ESC and iPSC	Trisomy 21	Not determined	Park et al. (2008), Biancotti et al. (2010)
Emanuel syndrome	iPSC	Supernumerary chr 11 attached to a piece of chr 22	Not determined	Li et al. (2012)
Familial amyotrophic lateral sclerosis (ALS)	ESC and iPSC	Mutations in *SOD1*, *VAPB*, *DPP6*, *IIPR2*, *IARDBP*, *FUS*	Downregulation of VAPB expression in fibroblasts, iPSCs, and motor neurons	Verlinsky et al. (2005), Dimos et al. (2008), Mitne-Neto et al. (2011)
Familial dysautonomia (FD)	iPSC	Mutation in *JKBKAP*	Splicing, cellular migration, and neurogenesis defects in FD-iPSC-derived neurons	Lee et al. (2009)
Fragile X syndrome (FXS)	ESC and iPSC	CGG triplet repeats in *FMR1*	Reduced expresion of *FMR1* through DNA methylation and histone modification Abnormal differentiation of FXS-iPSCs into neurons (fewer and shorter neurites)	Frumkin et al. (2010), Verlinsky et al. (2005), Eiges et al. (2007), Tropel et al. (2010)
Friedriech’s ataxia (FRDA)	iPSC	GAA triplet repeats in *FXN*	GAA triplet repeats in *FXN*, reduced *FXN* mRNA, defect in mismatch repair (MMR) enzymes in FRDA-iPSCs	Liu et al. (2011), Ku et al. (2010)
Huntington’s disease (HD)	ESC and iPSC	CAG triplet repeats in *HTT*	Increased susceptibility to growth factor withdrawal of HD-iPSC-derived NSCs Involvement of DNA mismatch repair (MMR) machinery in CAG instability	Tropel et al. (2010), Verlinsky et al. (2005), Park et al. (2008), Mateizel et al. (2006), Zhang et al. (2010)
Lesch–Nyhan syndrome	ESC and iPSC	Mutation in *HPRT1*	Not determined	Park et al. (2008), Urbach et al. (2004)
Neurofibromatosis type 1	ESC	Point mutation in *NF1*	Not determined	Verlinsky et al. (2005)
Parkinson’s disease (PD)	ESC and iPSC	Unknown or mutations in *LRRK2*, *PINK1*, *SNCA*, *PARK7*, *PRKN*, and others	Increased susceptibility to death for DA neurons derived from hESCs overexpressing the α-synudein Increased susceptibility to death for LRRK2-PD-iPSC-derived neurons when exposed to oxidative stress, proteasome inhibitor MG-132 and 6-hydroxydopamine Impairment of mitochondrial parkin recruitment and mitochondrial dysfuntion in PIMK1-PD-iPSC-derived DA neurons	Park et al. (2008), Soldner et al. (2009), Nguyen et al. (2011), Seibler et al. (2011), Devine et al. (2011), Schneider et al. (2007)
Patau syndrome	ESC and iPSC	Trisomy 13	Dramatic alterations in the expression of brain specific genes in ESC-derived EBs	Li et al. (2012), Biancotti et al. (2010)
Prader–Willi syndrome (PWS)	iPSC	15q11–13	Genomic imprinting of the imprinting center for PWS; reduced expression of the disease-associated small nucleolar RNA HBII-85/SNORD116	Chamberlain et al. (2010), Yang et al. (2010)
Retinopathies Retinitis pigmentosa	iPSC	Mutations in *RP1*, *RP9*, *PRPH2*, *RHO*, and others	Degeneration of RP-iPSC-derived rod photoreceptor cells Increase of apoptosis, oxidative stress and endoplasmic reticulum dysfunction in RP-iPSC-derived rod photoreceptor cells Identification of the cilia-related gene *male germ cell-associated kinase (MAK)* gene as a cause of RP	Jin et al. (2011), Tucker et al. (2011)
Gyrate atrophy		Mutations in *OAT*	Decline of ornithine-δ-aminotransferase activity; restored by vitamin B6 and via targeted gene repair	Meyer et al. (2011)
Rett syndrome (RTT)	iPSC	Mutation in *MECP2*	Morphological alterations of RTT-iPSC-derived neurons: fewer synapses, reduced dendritic spine density, and soma size Reduced frequency and amplitude of calcium transients and reduced frequency of spontaneous postsynaptic currents	Marchetto et al. (2010), Cheung et al. (2011), Kim et al. (2011c)
Schizophrenia (SCZD)	iPSC	Unknown	Reduced neuronal connectivity, outgrowth from soma, PSD95 dendritic protein levels in SCZD-iPSC-derived neurons Alterations of Notch signaling, cell adhesion, and slit-Robo-mediated axon guidance in SCZD-iPSC-derived neurons	Brennand et al. (2011), Chiang et al. (2011)
Spinal muscular atrophy (SMA)	iPSC	Mutation in *SMN1*	Absence of expression of SMN1, reduced number, and size of SMA-iPSC-derived motor neurons Deficit in neurite outgrowth and gem formation in SMA-iPSC-derived neurons	Ebert et al. (2009), Chang et al. (2011)
Spinocerebellar ataxia type2	ESC	CAG triplet repeats in *ATX2*	Not determined	Tropel et al. (2010)
Spinocerebellar ataxia type 3 or Machado–Joseph disease (MID)	iPSC	CAG triplet repeats in *ATX3*	Accumulation of ATX3 containing aggregates in MJD-iPSC-derived neurons, involvement of calpain, Na^+^ channels, K^+^ channels, ionotropic, and voltage-gated Ca^2+^ channels in the aggregate formation	Koch et al. (2011)
Warkany syndrome 2	iPSC	Trisomy 8	Not determined	Li et al. (2012)
X-linked adrenoleukodystrophy	ESC and iPSC	Mutations *in ABSCD1*	VLCFA accumulation in X-ALD-iPSC-derived oligodendrocytes; reduction of VLCFA levels in X-ALD-iPSC-derived oligodendrocytes by 4-phenylbutyrate and lovastatin	Verlinsky et al. (2005), Jang et al. (2011)

*ABCD1, adenosine triphosphate-binding cassette transporter superfamily D1 member; AD, Alzheimer’s disease; ALS, amyotrophic lateral sclerosis; AMN, adrenomyeloneuropathy form of X-linked adrenoleukodystrophy; APP, amyloid-β precursor protein; AS, Angelman syndrome; ATXN, ataxin; CAG, cytosine-adenine-guanine; CCALD, childhood cerebral form of X-linked adrenoleukodystrophy; CMT, Charcot Marie Tooth; CNS, central nervous system; DA, dopaminergic; DS, Down syndrome; EBs, embryoid bodies; ESCs, embryonic stem cells; FD, familial dysautonomia; FMR1, fragile X mental retardation; FRDA, Friedreich’s ataxia; FXD, fragile X syndrome; FXN, frataxin; HD, Huntington’s disease; HPRT, hypoxanthine-guanine phosphoribosyltransferase; HTT, huntingtin; iPSCs, induced pluripotent stem cells; LRRK2, Leucine-rich repeat kinase 2; MECP2, methyl CpG-binding protein 2; MJD, Machado–Joseph disease; NPCs, neural progenitor cells; NSCs, neural stem cells; OAT, ornithine-δ-aminotransferase; PD, Parkinson’s disease; PGD, pre-implantation genetic diagnosis; PINK1, PTEN-induced putative kinase 1; PS1 and PS2, presenilin 1 and presenilin 2; PSCs, pluripotent stem cells; PWS, Prader–Willi syndrome; RTT, Rett syndrome; SCZD, schizophrenia; SMA, spinal muscular atrophy; SMN1, survival motor neuron-1; SOD, superoxide dismutase; VAPB, vamp-associated protein B; VLCFA, very long chain fatty acid; X-ALD-iPSC, X-linked adrenoleukodystrophy*.

One year after the first reprogramming of human fibroblasts into hiPSCs (Takahashi et al., 2007; Ebert et al., 2009), Daley’s group reported the generation of several hiPSCs from patients affected by Mendelian or complex genetic disorders including the neurological diseases Gaucher’s disease, Parkinson’s disease (PD), HD, DS, and Lesch–Nyhan syndrome (Park et al., 2008). Since this first study, not a month goes by without a new article reporting the modeling of a human disease. Here, we report the principal neurodevelopmental and neurodegenerative diseases that have been modeled using hPSCs so far and the major findings regarding the pathogenesis of these diseases (Table 1).

#### Neurodegenerative diseases

##### Alzheimer’s disease

Alzheimer’s disease (AD) is the most common neurodegenerative disease. One in every eight of the population over 65 years old is estimated to have AD and 40–50% past the age of 85 may have it. AD is defined by progressive dementia with subsequent appearance of other cognitive, behavioral, and neuropsychiatric changes that degrade independence, social abilities of the affected patient in daily life. AD is characterized by neuronal and synaptic loss associated with extracellular deposits of amyloid-β peptides in senile plaques and intraneuronal neurofibrillary tangles (NFTs) formed by hyperphosphorylated tau (a microtubule-associated protein involved in microtubule stabilization; Querfurth and LaFerla, 2010). Most of AD forms are apparently sporadic (sAD) but dominantly inherited familial forms of AD (fAD) have been also reported; including those carrying mutation or duplication of *amyloid-*β *precursor protein* (*APP*) gene or mutations in the *presenilin 1* and *2* genes (*PS1* and *PS2*) which encode the major component of γ-secretase enzyme that cleaves APP into amyloid-β peptides and other cleavage fragments (Israel and Goldstein, 2011). At present, the study of AD pathogenesis is limited by the lack of access to live neurons from patients and the impossibility to model the sporadic form of AD. This limitation has been recently overcome by the generation of iPSCs from patients with sAD and fAD. Yagi and colleagues were the first reporting the generation and the characterization of iPSCs derived from fAD patients with mutations in *PS1* and *PS2* (fAD-iPSCs). In this study, fAD-iPSCs-derived neurons secreted more amyloid β42 in comparison with those from healthy donor, recapitulating the molecular pathogenesis of mutant presenilins (Yagi et al., 2011). More recently, Israel et al. derived iPSCs from two patients with fAD (fAD), both caused by a duplication of the amyloid-β precursor protein gene (fAD-iPSCs), two with sAD (sAD-iPSCs) and two non-affected individuals. The most striking results from this study are that fAD-iPSC- and sAD-iPSC-derived neurons exhibited significantly higher levels of the pathological markers amyloid-β(1–40), phospho-tau(Thr231), and active glycogen synthase kinase-3β (aGSK-3β) in comparison with those derived from healthy donors. Thus, they also accumulated large RAB5-positive early endosomes. Importantly, Phospho-Tau(Thr231) and aGSK-3β levels were reduced by treatment of the cells with β-secretase inhibitors (Israel et al., 2012).

##### Amyotrophic lateral sclerosis

Amyotrophic lateral sclerosis (also known as Lou Gehrig’s disease) is a fatal neurodegenerative disease characterized by injury and death of lower motor neurons in the brain stem and spinal cord, and of upper neurons in the motor cortex. The clinical hallmarks of ALS comprise the atrophy of skeletal muscle, eventual paralysis, respiratory failure, and death of patients within 1–5 years of disease onset. The incidence of ALS is two to three in 100,000 individuals. ALS is mostly a sporadic disease but 5–10% of cases are familial and usually of autosomal dominant inheritance. The pathogenic processes underlying ALS are multifactorial and are not completely known. In this regard, *superoxide dismutase 1* (*SOD1*), *peptidyl-peptidase 6* (*DPP6*), *inositol 1,4,5-trispohosphate receptor type 2* (*ITPR2*) and *Tar-DNA-binding protein-43* (*TARDBP*, also known as *TD43*), *fused sarcoma* (*FUS*), *vamp-associated protein B/C* (*VAPB*) have been identified as ALS susceptibility genes. Emerging evidence suggests that astrocytes and glia have an important role in the propagation of motor neuron injury in the sporadic and the familial forms of ALS (Glass et al., 2010; Ferraiuolo et al., 2011; Haidet-Phillips et al., 2011). Dimos et al. established iPSCs from an 82-year-old patient affected by a familial form of ALS with *SOD1* mutation. They showed that both normal iPSCs and ALS-iPSCs can differentiate into motor neurons but no phenotypic difference between the iPSC lines was reported (Dimos et al., 2008). New insights into the mechanisms underlying ALS degeneration have been gained when hESC-derived motor neurons were co-cultured with glial cells carrying a mutant allele of *SOD1* gene. Under these conditions, half of the hESC-derived motor neurons were lost whereas normal glial cells were not toxic. Prostaglandin and pro-inflammatory cytokines were found responsible for the toxic effect of these glial cells (Di Giorgio et al., 2008; Marchetto et al., 2010). At this step, studying glial cells derived from mutant SOD1-containing iPSCs (as the iPSC line reported in Dimos et al.) will provide crucial information on this toxicity: what render these cells toxic and what make the mutant SOD1-containing motor neurons more vulnerable than the normal ones? More recently, iPSCs were generated from ALS patients carrying mutation in *VAPB* gene, a susceptibility gene described as a rare cause of familial ALS. The protein encoded by *VAPB* gene is implicated in numerous cellular functions such as the regulation of lipid transport and homeostasis, formation of presynaptic terminal, and unfolded protein response (Ferraiuolo et al., 2011). The study did not reveal difference between the ALS-iPSCs and the normal ones in terms of their capacity to differentiate into motor neurons and regarding the intracellular distribution of VAPB protein upon basal condition and in the presence of MG-132 (a proteasome inhibitor that induces cytoplasmic inclusions of the VAPB protein). However, while the expression of VAPB protein constantly increased upon differentiation of the normal iPSCs into motor neurons, this expression remained significantly lower upon differentiation of the ALS-iPSCs (Mitne-Neto et al., 2011).

##### Familial dysautonomia

Familial dysautonomia (FD, Riley–Day syndrome, hereditary sensory, and autonomic neuropathy type III) is a rare neurodegenerative disease with autosomal recessive inheritance that occurs almost exclusively among individuals of Ashkenazi Jewish population. The disease affects the development and the survival of sensory, sympathetic, and some parasympathetic neurons. FD is caused by mutations in the *IKBKAP* gene, which encodes a protein called IKAP/hELP1 (IkB kinase complex associated protein). This mutation leads to a tissue-specific skipping of exon 20 of *IKBKAP* mRNA and subsequently to a reduced IKAP/hELP1 protein level in sensory and autonomic nervous systems. This protein has been shown to contribute to crucial processes within the cell such as actin cytoskeleton regulation, cell motility migration, acetylation of microtubules, and neuronal development. Recent advances have provided new insights into the underlying genetic and biochemical deficits in FD disease using iPSCs derived from patients with FD (Lee et al., 2009). Lee and colleagues derived iPSCs from three young patients affected by FD and differentiated them into neural cells. FD-iPSC-derived neural cells showed alterations in *IKBKAP* mRNA splicing, cell migration, and neurogenesis. Furthermore, the plant cytokinin kinetin corrected *IKBKAP* mRNA splicing and the neurogenesis defects but showed no effect on cell migration in these FD-iPSC-derived cells (Lee et al., 2009).

##### Huntington’s disease

Huntington’s disease is a severe late-onset autosomal dominant neurodegenerative disease that affects 5–7 in 100,000 Caucasian individuals. It is caused by CAG trinucleotide repeats in the exon 1 of the *huntingtin* (*HTT*) gene. The disease is characterized by the progressive loss of neurons, predominantly in the striatum, which leads to the typical motor, cognitive impairments, and dementia associated with the disease (Walker, 2007). Among the disease-specific iPSC lines generated in the early study of Park et al., iPSC lines were derived from a patient with HD (HD-iPSCs). DNA sequencing analysis of these HD-iPSCs confirmed the presence of 72 CAG trinucleotide repeats in one allele of *HTT* gene and 19 in the other (Park et al., 2008). Using the same HD-iPSC lines, Zhang et al. found an altered ERK activation when compared to normal iPSCs (Zhang et al., 2010), confirming previous reports (Apostol et al., 2006). Moreover, CAG trinucleotide repeats were conserved both after reprogramming of the HD-fibroblasts into HD-iPSCs and after the differentiation of the HD-iPSCs into neurons. The authors also documented their potential to differentiate into NPCs and to mature into striatal neurons but no phenotypic analysis was reported. Importantly, HD-iPSC-derived NSCs showed an increased susceptibility to growth factor withdrawal (Zhang et al., 2010). Also, HD-ESCs have been derived from embryos that harbor the mutant *HTT* allele by several groups (Mateizel et al., 2006; Niclis et al., 2009; Tropel et al., 2010; Bradley et al., 2011; Seriola et al., 2011). These HD-ESCs were pluripotent and showed the ability to differentiate into derivatives of the three germ layers *in vivo* and into NPCs *in vitro*. However, no phenotypic differences were reported (Bradley et al., 2011; Seriola et al., 2011). These studies also confirmed the presence of more than 40 CAG in these HD-ESCs (Bradley et al., 2011; Seriola et al., 2011) that remained stable upon differentiation (Seriola et al., 2011). Finally, the authors proposed that the downregulation of the proteins that form the DNA mismatch repair (MMR) machinery contributes to CAG instability in HD-iPSCs (Seriola et al., 2011). More recently, HD-iPSCs have been derived from homozygous and heterozygous HD patients. Importantly, both undifferentiated HD-iPSCs and HD-iPSC-derived neurons displayed a higher lysosomal activity compared to the normal counterparts (Camnasio et al., 2012).

##### Parkinson’s disease

Parkinson’s disease is a complex, multifactorial neurodegenerative disease of the basal ganglia and is recognized as one of the most common neurological disorders, affecting ~1% of individuals older than 60 years. There are two major neuropathological hallmarks: the loss of pigmented dopaminergic (DA) neurons in the substantia nigra and the presence of abnormal fibrillar cytoplasmic inclusions called Lewy bodies. It is unclear why neurons degenerate in PD but it is thought to be due to a combination of genetic and environmental factors (Dawson and Dawson, 2003). Indeed, although more than 90% of PD forms seem to be sporadic, a dozen of genes have been linked to the disease (Hardy, 2010). For example, multiplications of *SNCA* gene has been described in a highly penetrant and aggressive form of PD. This defect leads to α-synuclein protein aggregates in Lewy bodies (Hardy, 2010). Similarly, a common autosomal dominant missense mutation in *Leucine-rich repeat kinase 2* (*LRRK2*) gene is correlated with a penetrance of 85% in PD patients of 70 years old (Kachergus et al., 2005). Recessive inherited *Parkin* and *PTEN-induced putative kinase 1* (*PINK1*) mutations have been also described in PD cases with slowly progressive early onset disease (Hardy, 2010). Although animal models of PD have contributed indoubtfully to our current understanding of the disease, they fail to recapitulate PD pathogenesis accurately (Chesselet and Richter, 2011). The recent development of hPSCs provides a new method to create human cell-based disease model and to investigate the disease phenotype *in vitro*. Both hESCs and hiPSCs have been used for modeling PD condition. In an early report, Schneider and colleagues established hESCs that overexpressed the α-synuclein protein. An increased susceptibility to death of these cells was shown when differentiated into DA neurons (Schneider et al., 2007). More recently, iPSCs were derived from individuals with sporadic forms of PD (PD-iPSCs). However, from these studies it remains unknown whether PD-iPSC-derived neurons display a phenotype in comparison with the normal ones under basal condition (Park et al., 2008; Soldner et al., 2009; Nguyen et al., 2011; Seibler et al., 2011). Considering that PD-iPSC lines carrying the most common PD-related mutations may be appropriate to reveal and recapitulate key phenotypes of PD, two recent PD-iPSC models have been developed. The first one has been derived from patients with mutation in *LRRK2* gene (LRRK2-PD-iPSC). Importantly, this study revealed an increased expression of the α-synuclein protein and genes involved in oxidative stress when LRRK2-PD-iPSCs were further differentiated into DA neurons. Furthermore, LRRK2-PD-iPSC-derived neurons showed an increased susceptibility to cell death in comparison with the normal ones when exposed to oxidative stress, the proteasome inhibitor MG-132, and 6-hydroxydopamine (Nguyen et al., 2011). Similarly, iPSCs were derived from a PD patient carrying a triplication of *SNCA* gene (SNCA-PD-iPSCs) and an unaffected first-degree relative. When induced to differentiate into midbrain DA neurons, those derived from SNCA-PD-iPSCs showed a twofold increase of the α-synuclein protein expression, recapitulating the cause of disease phenotype of PD patients carrying this anomaly (Devine et al., 2011). More recently, iPSCs were generated from a PD patient harboring *PINK1* mutations. Under basal condition, no differences in the differentiation potential of the PINK1-PD-iPSCs into DA neurons were found when compared with normal ones. However, PINK1-PD-iPSC-derived DA neurons showed a ~5-fold reduction in *PINK1* mRNA levels. This study provides novel evidence for the role of *PINK1* mutations and the associated mitochondrial dysfunctions. In particular, contrary to DA neurons derived from normal iPSCs, mitochondrial depolarization of PINK1-PD-iPSC-derived DA neurons did not result in parkin protein translocation from the cytosol to mitochondria. This was accompanied by an increase of mitochondrial biogenesis as revealed by the increase of mitochondrial (mtDNA) copy number. The authors proposed that this increase could be explained by the induction of PGC-1α expression upon mitochondrial depolarization in PINK1-PD-iPSC-derived DA neurons (Seibler et al., 2011). Importantly, re-expression of parkin in PINK1-PD-iPSC-derived DA neurons corrected these defects (Seibler et al., 2011), supporting the crucial role of parkin protein in the pathogenesis of PINK1-linked PD.

##### Spinal muscular atrophy

Spinal muscular atrophy (SMA) is an inherited neuromuscular disorder caused by the mutation and/or deletion of the *survival motor neuron-1* (*SMN1*) gene. *SMN1* gene encodes the SMN protein, a protein found in the cytoplasm, and in nuclear bodies described as “gemini of coiled bodies” or gems. The disease is characterized by specific degeneration of alpha-motor neurons in the spinal cord, leading to muscle weakness, atrophy, and in the majority of cases, premature death. There are four forms of SMA that can be distinguished based on age of onset, pattern of muscle involvement, and inheritance pattern. Infants affected by the severe SMA (type I, Werdnig–Hofman disease) die before reaching the age of two, whereas the mild forms of the disease are characterized by relatively static muscle weakness for many years (Lunn and Wang, 2008). Ebert et al. derived iPSCs from a young boy affected by type I SMA and his unaffected mother. As expected, they confirmed the absence of SMN1 expression and the reduced presence of gems in SMA-iPSCs in comparison with the normal iPSCs. Interestingly, while no differences were found after 4 weeks of differentiation of the normal iPSCs and the SMA-iPSCs into motor neurons, SMA-iPSC-derived motor neurons were fewer and smaller than the normal ones, after 6 weeks of differentiation (Ebert et al., 2009). Treatment of the SMA-iPSCs with either valproic acid or tobramycin, two molecules that have been shown to increase SMN protein levels, efficiently increased the expression of SMN protein as well as gems in the treated cells. However, the effects of these molecules in motor neurons were not addressed. More recently, SMN protein re-expression in SMA-iPSCs restored neurite outgrowth and gem formation deficits (Chang et al., 2011). Taken together, these two studies provide the proof of principle that SMA-iPSCs can be used to model the disease and that it is possible to improve the phenotype using both pharmacological and gene correction approaches.

##### Spinocerebellar ataxia

Spinocerebellar ataxia is an inherited disorder of brain function with at least 28 distinct genetic forms. Patients affected by the disease experience a degeneration of the spinal cord and the cerebellum. All types of spinocerebellar ataxia are characterized by a progressive incoordination of walking and are often associated with poor coordination of hand movements, eye movements, and speech (Paulson, 2007). Machado–Joseph disease (MJD, also called spinocerebellar ataxia type 3) is the most common spinocerebellar ataxia. This neurodegenerative disease is caused by expansion of CAG triplet repeats in the *MJD1* gene (also called *ATXN3*, *ataxin-3*). The neuropathological hallmark of MJD patients is the accumulation of ATXN3 protein-containing aggregates in brain tissue; the severity of the disease is directly correlated with the amount of such aggregates. Even if the gene and the anomalies are known, the pathogenic mechanisms underlying these abnormalities remain not well understood (Costa and Paulson, 2012). ESCs have been derived from embryos that harbor the mutant *SCA2* gene (also called *ATXN2*, *ataxin-2*; Tropel et al., 2010), however to our best knowledge, no studies have been conducted using these cells. Recently, iPSCs were derived from four patients affected by MJD (MJD-iPSCs) and two related healthy donor. As expected, expansion of polyQ-coding CAG sequence in *MJD1* gene was verified in MJD-iPSCs. However, no differences were found with respect to the differentiation potentials and the functional properties between the MJD-iPSC-derived neurons and those from healthy donors. Importantly, upon repetitive stimulations with l-glutamate or *N*-methyl-d aspartate (NMDA), MJD-iPSC-derived neurons accumulated ATXN3 protein-containing aggregates whereas those from healthy donors did not. This aggregate formation was shown to involve the recruitment of other polyQ proteins (such as the TATA binding protein) and the calcium-dependent activation of caspase and calpain proteases (Koch et al., 2011).

##### X-linked adrenoleukodystrophy

X-linked adrenoleukodystrophy is a neurological disorder that occurs most often in males. It mainly affects the nervous system and the adrenal glands. There are three distinct types of X-ALD: a severe early onset childhood cerebral form (CCALD), an adrenomyeloneuropathy form (AMN), and a type called “Addison disease only.” CCALD manifests between the age of 4 and 8 years and is characterized by attention deficit, progressive impairment of cognition, behavior, vision, and motor function that often lead to total disability within 2 years. AMN is a more slowly progressive form that manifests in adult life as progressive paraparesis, sphincter disturbances, sexual dysfunction, and often, impaired adrenocortical function. In contrast, the “Addison disease only” is a variant without neurological involvement. Female carriers present milder phenotype than males; they develop neurologic manifestations close to the AMN form with a later onset. The disorder is caused by mutations in the *adenosine triphosphate-binding cassette transporter superfamily D1 member* (*ABCD1*) gene that encodes ABCD1 protein (or ALDP), a peroxisomal protein necessary for beta-oxidation of very long chain acids (VLCFA) in the peroxisomes. As a result, elevated VLCFA levels accumulate in plasma and tissues together with the loss of axons and the demyelination in the long tracts of the spinal cord. At present, even the gene responsible for X-ALD is known, the mechanisms by which VLCFA accumulation in tissues leads to the neurological defects remain unknown (Ferrer et al., 2010). A recent study using iPSC technology opened a new avenue for the study of X-ALD pathogenesis. Jang et al. generated iPSCs from patients with CCALD (CCALD-iPSCs) and AMN (AMN-iPSCs). Both iPSCs displayed mutations in the *ABCD1* gene. Considering that the cerebral demyelination resulting from oligodendrocyte degeneration and the loss of neurons are the two major hallmarks of X-ALD, CCALD-iPSCs, and AMN-iPSCs were differentiated into neurons and oligodendrocytes. No difference was found in the differentiation potentials of CCALD-iPSCs and AMN-iPSCs when differentiated into neurons and oligodendrocytes in comparison with the normal ones (Jang et al., 2011). These results are consistent with the absence of developmental defect observed in the brain of X-ALD patients before onset of the disease (Ferrer et al., 2010). However, VLCFA levels were greater in neurons and oligodendrocytes derived from CCALD-iPSCs and AMN-iPSCs in comparison with the normal counterparts. Thus, VLCFA levels were significantly higher in CCALD-iPSC-derived oligodendrocytes compared with AMN-iPSC-derived ones recapitulating the much more severe phenotype observed in the CCALD form. Moreover, VLCFA levels in CCALD-iPSC-derived oligodendrocytes were significantly reduced by 4-phenylbutyrate and lovastatin, two compounds that upregulate the expression of *ABCD2*, a closely related *ABCD1* gene that probably compensates the *ABCD1* gene defects. The reduction of VLCFA levels in CCALD-iPSC-derived oligodendrocytes by pharmacological approaches gives the proof of principle that these iPSCs provide a promising model not only to study the pathogenesis of the disease but also to test compounds that restore the disease phenotype (Jang et al., 2011).

#### Neurodevelopmental diseases

##### Angelman syndrome

Angelman syndrome (AS) is a neurodevelopmental disorder with an estimated incidence between 1 in 10,000 and 1 in 20,000 individuals. AS is characterized by severe mental retardation, neurological problems, absence of speech, dysmorphic facial features, microcephaly, epileptic seizures, and electroencephalogram abnormalities. It is caused by a variety of genetic abnormalities involving the chromosome 15q11–13 region (60–75%), paternal uniparental disomy (2–5%), imprinting defect (2–5%), and mutation in the *ubiquitin protein ligase E3A* (*UBE3A*) gene (10%). *UBE3A* is subjected to a tissue-specific genomic imprinting. The paternally inherited allele is repressed and the maternally one is expressed in mature neurons of the brain whereas both alleles are expressed in the remaining tissues (Van Buggenhout and Fryns, 2009). *UBE3A* imprinting is thought to be mediated by a long non-coding transcript called *UBE3A-ATS* in human. Mouse models of AS exist but differ from human condition in the timing, mechanisms, and tissue specificity of *UBE3A* repression (Leung et al., 2011). In a recent study, Chamberlain et al. established iPSC lines from two patients with AS who carried maternally inherited deletions of chromosome 15q11–q13 (AS-iPSCs). AS-iPSCs maintain the methylation imprint of the parental fibroblasts following reprogramming and after long term culture. This iPSC-based model recapitulates the tissue-specific pattern of *UBE3A* imprinting as the paternal *UBE3A* was silenced in AS-iPSC-derived neurons in contrast with the normal ones. The authors demonstrated that *UBE3A* silencing is mediated by the sudden expression of *UBE3A-ATS* during neurogenesis (Chamberlain et al., 2010). Considering the results of this study, this iPSC-based model could allow a better understanding of the mechanisms that govern genomic imprinting during human neural development in AS.

##### Down syndrome

Down syndrome is the most common genetic developmental disorder with an incidence of 1 in 800 live births. It is caused by a trisomy of the chromosome 21 and results in varying degree of physical and mental retardation. With respect to the mental disturbances, patients with DS show cognitive impairment, learning and memory deficits, arrest of neurogenesis, and synaptogenesis and early onset of AD (Antonarakis et al., 2004). Recently, hESCs have been identified by PGD from human embryos that carried trisomy 21 anomaly (DS-hESCs). When induced to differentiate as EBs, the DS-hESC-derived cells displayed chromatin modifications in comparison with the normal counterpart (Biancotti et al., 2010). iPSCs have been also derived from patients with DS (Park et al., 2008) but their neural differentiation potentials remain still not investigated.

##### Fragile X syndrome

The neurodevelopmental disorder FXS is the most common cause of intellectual disability in males and the most common single gene cause of autism. In addition to cognitive deficits, FXS patients exhibit hyperactivity, attention deficits, social difficulties, anxiety, and other autistic-like behaviors. This X-linked disorder is caused by an expansion of trinucleotide CGG repeats on the promoter region of the *fragile X mental retardation 1* (*FMR1*) gene that leads to the loss of the fragile X mental retardation protein (FMRP). The first PSCs reported for the study of FXS were derived from embryos identified by PGD (Eiges et al., 2007; Tropel et al., 2010). Eiges and colleagues established an FXS-hESC-based model for the study of the developmental events involved in the pathogenesis of the disease. The full expansion in CGG repeats was not able to inactivate the expression of *FMR1* gene in the undifferentiated FXS-hESCs. However, upon *in vivo* differentiation, FMR1 expression was significantly down-regulated through epigenetic silencing which involves DNA methylation and histone modifications (Eiges et al., 2007). More recently, FXS-iPSCs were generated from patients affected by FXS but interestingly, these cells do not confirm the differentiation dependent silencing of *FMR1* gene expression observed in FXS-hESCs (Urbach et al., 2010). Using FXS-iPSCs, Sheridan et al. (2011) provide novel evidence that the epigenetic modifications of *FMR1* gene together with the loss of FMRP expression is responsible for the abnormal differentiation and maturation of FXS-iPCs into neurons.

##### Friedreich’s ataxia

Friedreich’s ataxia (FRDA) is the most frequent hereditary ataxia, with an estimated prevalence of three to four cases per 100,000 individuals. This autosomal recessive neurodegenerative disease is characterized by progressive gait and limb ataxia, dysarthria, areflexia, loss of vibration sense, and a progressive motor weakness. GAA triplet repeat expansions within the first intron of the *frataxin* (*FXN*) gene are the most common mutations underlying FRDA. As a consequence, patients show reduced levels of a *FXN*-encoded mitochondrial protein called frataxin. The subsequent mitochondrial dysfunctions in neuronal and muscle cells lead to degeneration of nerve tissue in the spinal cord and nerves controlling muscle movement in the arms and legs. Non-neurological signs include hypertrophic cardiomyopathy and diabetes mellitus. Mouse models for FRDA and FRDA cell lines are readily available, however they do not accurately mimic the disease (Schulz et al., 2009). In two recent reports, iPSCs were successfully derived from patients with FRDA (FRDA-iPSCs). *FXN* mRNA levels were significantly reduced in the FRDA-iPSCs and FRDA-iPSC-derived EBs and NPCs. In addition, FRDA-iPSCs showed the characteristic GAA triplet repeat expansions in the *FXN* gene (Ku et al., 2010; Liu et al., 2011). The mechanistic analysis of these GAA repeat expansions revealed the involvement of the MMR enzymes MSH2 in the repeat instability observed in FRDA-iPSCs. Moreover, global mRNA expression profile analysis of FRDA-iPSCs points to a role for genes related to mitochondrial function, DNA repair, DNA damage response, cell cycle, protein modification/ubiquitination, lipid metabolism, and carbohydrates biosynthesis, confirming previous results found in FRDA patients (Ku et al., 2010). Further differentiation of FRDA-iPSCs into sensory neurons will advance the understanding of the impact of GAA repeat expansions in the dysfunction and death of the sensory neurons of the dorsal root ganglia in FRDA patients.

##### Prader–Willi syndrome

Prader–Willi syndrome (PWS) is a neurodevelopmental disorder caused by a deletion or disruption of genes in the proximal arm of chromosome 15 or by maternal uniparental disomy in the proximal arm of chromosome 15 (also called critical 15q11–13 region). In addition to mental retardation, PWS is characterized by reduced fetal activity, obesity, hypotonia, short stature, hypogonadotropic hypogonadism, small hands, and feet. PWS is frequently described together with AS because both are caused by genomic imprinting of the critical 15q11–13 region. The disease is due to genomic imprinting on the critical chromosomal region where the expression of genes from only one parent’s chromosome is associated with silencing of those from the other parent’s chromosome. The imprinting center (IC) for PWS is located in the exon 1 of the *SNURF-SNRPN* gene. This IC seems to act as a promoter for *SNURF-SNRPN* and the small nucleolar RNAs (snoRNA) *HBII-85* (also called *SNORD116*) and *HBII-52* (also called *SNORD115*) genes (deficiency of these snoRNAs is sufficient to cause PWS). As a consequence, the PWS IC of paternal origin is normally demethylated whereas the high methylation of the maternal PWS IC leads to the silencing of *SNURF-SNRPN* gene (Leung et al., 2011). Recent advances have been achieved by modeling PWS through the generation of iPSCs from individuals affected by PWS (PWS-iPSCs; Chamberlain et al., 2010; Yang et al., 2010). These PWS-iPSCs expressed markers of pluripotency, showed DNA hypomethylation of *Nanog* and *Oct4* promoters and were able to differentiate *in vivo* and *in vitro* into the three germ layers. Importantly, PWS-iPSCs maintained an appropriate methylation imprint after reprogramming. In contrast with the normal iPSCs where a methylated maternal allele and an unmethylated paternal allele was present, PWS-iPSCs showed only a methylated maternal allele (Chamberlain et al., 2010). In addition, PWS-iPSCs retained the genomic imprinting of the parental fibroblasts for PWS IC and showed a silencing of *HBII-85* gene expression (Yang et al., 2010).

##### Rett syndrome

Rett syndrome (RTT) is a neurological disorder caused by mutations in the X-linked gene *methyl CpG-binding protein 2* (*MECP2*). It affects almost exclusively females as young boys inheriting a mutant *MECP2* are much more severely affected and usually do not survive after infancy. It is the primary cause of severe mental retardation in girls with an incidence of ~1 in 10,000 female births (Neul et al., 2010). Recently, RTT disease phenotype has been successfully recapitulated in RTT-iPSC-derived neurons. In particular, when RTT-iPSCs were induced to differentiate into neurons, they displayed morphological alterations such as fewer synapses, reduced dendritic spine density, and soma size (Marchetto et al., 2010; Cheung et al., 2011; Kim et al., 2011c). Thus, electrophysiological recordings revealed a decrease of the frequency and the amplitude of calcium transients together with a reduced frequency of spontaneous postsynaptic currents in RTT-iPSC-derived neurons, supporting the idea that calcium signaling is impaired in these cells (Marchetto et al., 2010). The same group provided novel evidence into the mechanisms underlying the pathogenesis of RTT disease. They found in particular that long interspersed nuclear elements-1 (LINE-1 or L1s) retrotransposition, a process that modulates gene expression through insertions, deletions, and newsplice sites, is more frequent in RTT-iPSC-derived neurons than those derived from normal healthy donors (Muotri et al., 2010).

##### Schizophrenia

Schizophrenia (SCZD) is a heritable developmental disorder that affects ~0.5–1% of the population. This psychiatric disorder is characterized by psychotic symptoms (hallucinations, delusions, disorganized speech, and behavior), negative symptoms (flattened affect, avolition, and social withdrawal), and cognitive defects. Typically, patients with SCZD show decreased brain volume, aberrant neurotransmitter signaling, reduced dendritic arborization, and impaired myelination. Chiang et al. first published the generation of iPSCs from SCZD patients with a mutation in *Disrupted-in-Schizophrenia-1* (*DISC1*), a susceptibility gene that have been previously described disrupted in Finnish SCZD families (Ekelund et al., 2001). However, the neural differentiation potentials and the functional properties of these SCZD-iPSCs were not investigated in this study. Insights into the pathogenesis of SCZD have been gained by direct reprogramming of fibroblasts from patients affected by SCZD into SCZD-iPSCs and subsequent differentiation of these iPSCs into neurons (Brennand et al., 2011). SCZD-iPSC-derived neurons had reduced neuronal connectivity, reduced outgrowths from soma and reduced PSD95 dendritic protein levels. Thus, the authors not only confirmed the alteration of genes known to be involved in the pathogenesis of SCZD but also updated new altered pathways in SCZD. Importantly, these defects in neuronal connectivity and gene expression were ameliorated by the antipsychotic drug loxapine (Brennand et al., 2011). Taken together, these results support the idea that disease-specific iPSCs not only allow the investigation of the mechanisms involved in the pathogenesis but also the restoration of the defects associated with the disease.

#### Retinal degenerative diseases

Retinitis pigmentosa (RP) is the most common inherited human eye disease (with a worldwide prevalence of 1 case in 3000 to 1 in 7000 individuals) caused by the irreversible degeneration of rod photoreceptors. This results in night blindness and visual defects that can lead to complete blindness when the disease further affects the cone photoreceptors. The mechanisms underlying retinal degeneration are largely unknown; hundred of genes have been associated with the disease and therefore clear genotype-phenotype correlations are not possible (Ferrari et al., 2011). Recent advances in stem cell technology have led to the emergence of methods for differentiation of PSCs into multipotent retinal progenitor cells (RPCs), retinal pigment epithelium (RPE), and photoreceptor-like cells (Buchholz et al., 2009; Meyer et al., 2009, 2011; Osakada et al., 2009; Lamba and Reh, 2011). In addition, disease-specific iPSCs have been derived from patients affected by RP (RP-iPSCs) carrying mutations in *RP1*, *RP9*, *PRPH2*, or *RHO* genes (Jin et al., 2011; Tucker et al., 2011). Interestingly, in contrast with their normal counterpart, RP-iPSC-derived rod photoreceptor cells degenerated with extended culture period. The authors provide evidence that this degeneration was triggered by an increase of apoptosis, oxidative stress, and endoplasmic reticulum dysfunction in these cells (Jin et al., 2011). Importantly, the degeneration of rod photoreceptors carrying *RP9* mutations was counteracted by the antioxidant α-tocopherol but not in those carrying *RP1*, *PRPH2*, or *RHO* mutations supporting the idea that the efficacy of the molecule depends on the genetic mutations (Jin et al., 2011). In an other study, the genetic analysis of the RP-iPSCs lead to the identification of the cilia-related gene *male germ cell-associated kinase* (*MAK*) gene as a cause of RP (Tucker et al., 2011). Similarly, iPSCs has been established from patients affected by gyrate atrophy, an autosomal recessive eye disease characterized by progressive loss of vision due to retinal degeneration. The affected iPSC-derived RPE exhibited disease-specific functional defects (such as a profound decline of ornithine-δ-aminotransferase activity) that could be restored pharmacologically using vitamin B_6_ and via targeted gene repair (Meyer et al., 2011). Altogether, these studies strongly support the idea that these iPSC-based models provide a promising opportunity to identify the pathogenic mechanisms involved in retinal degeneration and give the proof of principle of functional correction of the disease phenotype using both pharmacological and gene repair approaches.

### Drug screening/toxicity

Even if progress has been made in pharmacological treatment of some neurological diseases, most of them have minor supportive therapy to no cure available. Moreover, drug development is an incredibly expensive and time consuming process. Discovering and bringing one new drug to the public typically costs from $800 million to more than $1 billion and takes an average of 10–15 years for a pharmaceutical company. The vast majority of the candidate molecules by the current drug screening methods fails to become a drug in clinical application because of safety and efficacy issues. In other terms, current drug screening methods are insufficiently predictive for clinical toxicity and efficacy. There are many explanations accounting for this. First, the main human cellular models used for drug discovery are primary cells isolated from patient tissue and transformed cells derived from tumors or genetically modified. Even if notable insights have been gained with these cells, the limited availability and the relevance of these cells reduce their potential for drug discovery. Then, despite similarities to human patient’s phenotype (Baker, 2011), mice models have several drawbacks for disease modeling and drug screening (Dibernardo and Cudkowicz, 2006; Scott et al., 2008). Perhaps the best example is the use of the transgenic mouse that overexpresses mutant superoxide (SOD), a gene found to be associated with ALS (Rosen et al., 1993). Several compounds including vitamin E and creatine were beneficial in this mouse model (Klivenyi et al., 1999) but showed no clinical improvement in humans (Desnuelle et al., 2001; Shefner et al., 2004; Aggarwal and Cudkowicz, 2008; Schnabel, 2008). Therefore, there is a real need to more accurately model human physiology. In this context, hESCs and hiPSCs provide a unique opportunity for drug discovery (Figure 2). In fact, after the identification of the targets involved in the pathogenesis of the disease, the next step could be the targeting of the defects using pharmacological and gene correction approaches. As a proof of concept, numerous recent studies using hESCs and hiPSCs began with target identification by choosing a biochemical mechanism involved in a disease condition, followed by the rescue of the observed defects. Defect corrections have been reported in hiPSCs with known drugs that have been previously reported beneficial in SMA (Ebert et al., 2009), FD (Lee et al., 2009), SCZD (Brennand et al., 2011), AD (Israel and Goldstein, 2011), and retinopathy (Meyer et al., 2011). By using hiPSCs, it is not only possible to confirm the interaction of the candidate molecules with the drug target, but also allows the evaluation of their efficacy by checking their activity in the neural cell of interest regarding the disease. Thereafter, the potential of the drug candidate can be assessed by rigorous screening processes which can include functional genomics and/or proteomics as well as other functional screening methods. Also, the hiPSC model offers the obvious possibility of personalized screening of molecules. By using patient specific-iPSC-differentiated cells, it could be possible to test and adapt the dose and combination of treatments to the patient. At the same time, it allows the exploration of the possible targets of patient resistance to treatments.

A critical issue for clinical translation is safety. For example, some drugs which are not aimed at targeting heart or liver have nevertheless been found to have profound toxic effects on heart muscle and hepatocyte. Cardiotoxicity and hepatotoxicity are the major forms of toxicity seen in drug development. Safety issues can be tested at earlier stage using hPSC-derived cells. Screening of the hepatotoxic and cardiotoxic effects of drugs can be evaluated by directed differentiation of the hPSCs into hepatocytes and cardiomyocytes. Similarly, due to their reliance on embryonic and differentiation pathways, hPSCs are potentially informative for embryonic development and differentiation screens (Desbordes et al., 2008). These screens may identify molecules involved in cell specification and toxicity pathways in embryonic development and differentiation of hPSCs.

### Regenerative medicine: From developmental biology to therapeutic applications

Perhaps the most important potential application of hPSCs is the generation of cells and tissues that could be used for cell-based therapies (Figure 2). The possibility to replace lost neurons or other neural cell types and to support the remaining neural cell population by hPSC-derived cells has received considerable attention. Cell replacement may be achieved by transplantation into patients of hPSC-derived cells which have undergone differentiation and maturation *in vitro*. Preliminary research in animal models indicates that hPSC-derived cells, transplanted into a damage brain or retina, can have beneficial effects. Whether these cells can generate the neural cells of interest (neurons, glial cells, and RPCs) or stimulate the endogenous stem cells in the CNS that repopulate the damage tissue is actively under investigation. Proof of principle for such regeneration has been demonstrated for several CNS disease models. In this section, we will discuss four striking breakthroughs of PSCs in regenerative medicine.

Regarding spinal cord injury, PSC-derived cells are currently used to replace the damage area or to support axonal growth with trophic factors. Transplantation of hESC-derived neurospheres, motor neurons, or oligodendrocytes in rodent models of spinal cord injury has been shown to improve function. These hESC-derived oligodendrocytes have been shown to repopulate the site of injury and promote remyelination of the lesion (Keirstead et al., 2005; Lee et al., 2007b; Nori et al., 2011). Based on the impressive results published in animal models of spinal cord injury, the U.S. Food and Drug Administration (FDA) has approved Geron Corp. for human clinical trials using hESC-derived oligodendrocyte progenitors (GRNOPC1) in spinal cord injury (studies registered with ClinicalTrials.gov, number NCT01217008). GRNOPC1 were administered by injection at a dose of two million cells between 7 and 14 days after injury in four patients with complete thoracic spinal cord injuries. To date, GRNOPC1 has been well tolerated with no serious adverse events observed.

Motor neuron degeneration is a pathological hallmark of motor neuron diseases such as ALS and SMA for which currently no cure exists. Recently, motor neuron replacement and protection using hPSCs has emerged as potential candidates for the treatment of motor neuron diseases. To be clinically successful, the transplanted hPSC-derived cells have to form extended axons and functional neuromuscular junctions. In rat models, spinal transplantation of hESC-derived motor neuron progenitors has resulted in partial recovery from paralysis thanks to axonal projection and muscle innervation (Harper et al., 2004; Deshpande et al., 2006; Corti et al., 2009, 2010).

Cellular therapy for PD remains quite challenging. The disease results from the degeneration of DA neurons in the substantia nigra and the subsequent loss of dopamine in the striatum. Initial studies investigated the potential of hESC-derived DA neurons in rodent models of PD. Roy et al. (2006) documented the functional engraftment of hESC-derived DA neurons together with improvement of lesion-induced behavioral deficits in a rodent model of PD. Thereafter, numerous studies supported the clinical potential of hPSCs for personalized cell therapy of PD (Tabar et al., 2008; Wernig et al., 2008b; Hargus et al., 2010; Rhee et al., 2011). The recent study of Studer and colleagues represents a major advance toward the application of hESC-derived DA neurons in clinic. They succeeded in generating DA neurons with a substantia nigra phenotype from hESCs that exhibited electrophysiological properties of substantia nigra neurons and released DA *in vitro*. Notably, these cells demonstrated *in vivo* survival and function when transplanted in three animal models of PD. In 6-hydroxy-dopamine-lesioned mice and rats, these DA neurons functionally engrafted *in vivo*, reinnervated the striatum and improved clinically relevant behavioral deficits resembling symptoms in PD patients. Importantly, the authors did not identify any neural overgrowth or tumors of the transplanted neural cells *in vivo* supporting a future hESC-based therapy for PD patients (Kriks et al., 2011).

Another important area of investigations for hPSCs is cell-based therapy for retinal degenerative diseases such as retinitis pigmentosa, gyrate atrophy, and age-related macular degeneration. The successful differentiation of hPSCs into multipotent RPCs, RPE, and photoreceptor-like cells (Buchholz et al., 2009; Meyer et al., 2009, 2011; Osakada et al., 2009; Lamba and Reh, 2011) has opened new hopes and perspectives for the therapy of retinal degenerative diseases. In the past few years, promising studies with transplantation of hPSC-derived cells in animal models of retinal degeneration have caused great excitement. In particular, hESC-derived RPE cells provided long term rescue of visual function in two rodent models of retinal degeneration, by replacing the degenerating retina (Gamm et al., 2007; Francis et al., 2009; Lu et al., 2009). Thus, the FDA has granted the permission to Advanced Cell Technology’s for clinical trials using hESC-derived RPE (MA09-hRPE cells) for Stargardt macular dystrophy (SMD) and dry age-related macular degeneration (AMD; studies registered with ClinicalTrials.gov, numbers NCT01345006 and NCT01344993). A preliminary report regarding the safety and tolerability of this trial in one patient with AMD and the other with SMD showed no signs of hyperproliferation, tumorigenicity, ectopic tissue formation, or immune rejection of the hESC-derived RPE cells 4 months after transplantation (Schwartz et al., 2012).

## Challenges and Limits

As hESCs are derived from embryos, their use for clinical application and basic research remains controversial. In addition to the obvious technical and ethical considerations about the use of hESCs, one of the major barriers for their clinical use is the challenge of immunological rejection (for a review regarding the immunological aspects of PSCs see de Rham and Villard, 2011; Preynat-Seauve and Krause, 2011). In this regard, the iPSCs provide an alternative source of autologous stem cells. Moreover, iPSCs do not require the use of human embryos or oocytes, which makes their use in basic research and in clinical application less controversial technically and ethically. Despite those advantages, significant barriers, and challenges remain unsolved in their current use in research and before their applications in clinic.

### Disease modeling

Pluripotent stem cells have opened a new door to study and understand human diseases. However, it is important to keep in mind that it is not possible to model all human neurological diseases *in vitro* using PSCs. The lack of hESC for some diseases that cannot be identified after PGD, account for this (see Table 1 for the diseases in which ESCs have been derived from human embryos). This limitation concerns also iPSCs. One recent exception is cells derived from patients affected by FXS which failed to reactivate the *fragile X mental retardation 1* (*FMR1*) gene after reprogramming into iPSCs. In contrast, ESCs derived from human FXS blastocysts showed the reactivation of the *FMR1* gene (Urbach et al., 2010). This example suggests that iPSCs may not be the model of choice to study certain human genetic diseases. Moreover, even though a disease-related phenotype has been shown with iPSCs derived from patients with FD, SMA, RTT, and others (see the section “modeling human neurological diseases”), in contrast a phenotype has not been found *in vitro* using iPSCs derived from patients with PD (PD-iPSCs) and HD under basal conditions (Park et al., 2008; Soldner et al., 2009; Nguyen et al., 2011; Seibler et al., 2011). In fact, FD, SMA, and RTT manifest early in life and therefore are more prone to show the disease phenotype *in vitro* using iPSCs. Many common human neurological diseases have late-onset like AD and PD. So, a key challenge is to produce PD-iPSC-derived cells with the neuron characteristics of a 75-year-old patient affected by PD. In this regard, it remains unclear whether iPSCs retain an epigenetic memory and age-related behavior of the parental somatic cells. If yes, this could allow the modeling at least in part, of late-onset diseases. If not, iPSC-derived neural cells may not manifest the phenotype under basal conditions. Furthermore, it could be also possible to induce the age-related phenotype pharmacologically (using free radicals, molecules that induce aging, and neurodegeneration) or by gene manipulation (mitochondrial DNA mutations). As an example, Nguyen et al. derived iPSCs from a patient with a mutation in the *LRRK2* gene, the most common cause of familial PD. Interestingly, DA neurons derived from these LRRK2-PD-iPSCs displayed a greater susceptibility to cell death when exposed to stress agents such as oxidative stress, the proteasome inhibitor MG-132, or 6-hydroxydopamine (Nguyen et al., 2011). Similarly, in DA neurons derived from PD-iPSCs harboring *PINK1* mutations, an impairment of the mitochondrial parkin recruitment has been described upon mitochondrial depolarization induced by valinomycin (Seibler et al., 2011). Neurodegeneration can also be induced by reproducing the toxic microenvironment of the dying cells. In an elegant study, neurodegeneration in ALS has been recapitulated by co-culture of hESC-derived motor neurons with glial cells carrying SOD mutations (Di Giorgio et al., 2008; Marchetto et al., 2008). Therefore, co-culture of glial cells with motor neurons derived from ALS-iPSCs carrying SOD mutations will be of great interest for the understanding of the role of the glial cells in motor neuron degeneration.

A number of potential variables must be considered when establishing an hPSC-based disease model. Regarding disease modeling and drug screening studies, the definition of a non-disease control is of crucial importance (Inoue and Yamanaka, 2011; Zhu et al., 2011). First and foremost, the genetic background of the non-disease control and the affected cells has to be identical or close in order to be sure that the differences observed in the studies are only due to the disease and not to the choice of the normal and the affected samples. In practice, most of the published articles used iPSCs from unaffected family members of the patient as controls. When this condition is not possible to achieve, control iPSCs from unrelated healthy persons together with ones from unrelated affected patients are often used to decrease the variability between the control and the affected cells and to ensure that the results are not specific for a particular control and patient. To overcome these problems isogenic controls have been recently developed using several approaches. For example, recent studies have described the possibility to obtain isogenic controls through X-chromosome inactivation as after reprogramming, iPSCs can retain an inactive X-chromosome in a non-random pattern. Taking advantage of this characteristic, several groups obtained a pair of isogenic wild-type and mutant iPSC lines. One example was the generation of a pair of isogenic normal iPSCs and mutant MECP2 expressing RTT-iPSCs (Ananiev et al., 2011; Cheung et al., 2011). Then, for monogenic diseases, isogenic controls can be generated through targeted correction of genetic point mutations. One strategy for correction is to use homologous recombination with an exogenous DNA to modify specific genomic sequences. This is referred as “genome editing” and comprises the engineered zinc finger nucleases (ZFNs), transcription activator-like effector nucleases (TALENs), and oligonucleotide-directed gene editing methods (Lombardo, 2007; Miller, 2007; Moehle, 2007; Hockemeyer et al., 2011). The principal advantage of ZFNs is the ability to target any desired genomic DNA sequence with high fidelity and to induce precise gene knockouts or gene replacements by homologous recombination. This approach has been recently applied to target endogenous genes in hESCs and hiPSCs to generate isogenic disease and control cell lines (Hockemeyer et al., 2009; Soldner et al., 2009; Zou et al., 2009). The genetic corrections of the sickle cell anemia mutation (Sebastiano et al., 2011) and of the α1-antitrypsin deficiency (Yusa et al., 2011) in hiPSCs are examples of recent accomplishments using this technology.

Other factors are likely to contribute to the variability between iPSC lines such as the process of cell derivation (Lengner et al., 2010). Considering that iPSCs were found to retain epigenetic memory of their parental somatic cells and showed preferential lineage-specific differentiation (Bar-Nur et al., 2011; Kim et al., 2011b), it is important to take the same type of parental somatic cells when establishing iPSCs. Moreover, the residual expression of the viral vector (Soldner et al., 2009), the genetic alterations introduced after reprogramming (Gore et al., 2011; Hussein et al., 2011), and the protocols used for differentiation (either spontaneous differentiation into EBs or directed differentiation into neural cells of interest) may contribute to the observed variations in efficiency between iPSC clones in generating neural cells (Hu et al., 2010).

### Regenerative medicine

The hPSC applications in regenerative medicine are an exciting and fast moving area of current studies. The recent findings are supportive of a future hPSC-based therapy for neurological diseases. Long term engraftment of hPSC-derived cells in several CNS disease models demonstrated *in vivo* survival and function of these cells together with improvement up to complete restoration of the deficits resembling the symptoms observed in human neurological diseases (Harper et al., 2004; Keirstead et al., 2005; Deshpande et al., 2006; Gamm et al., 2007; Corti et al., 2009, 2010; Francis et al., 2009; Lu et al., 2009; Kriks et al., 2011; Schwartz et al., 2012). Also, hPSCs offer the advantage to provide an inexhaustible supply of differentiated cell types compared to the other cells that have been used in clinic until now (mesenchymal stem cells, fetal, and adult stem cells). Another important advantage of hPSCs for regenerative medicine is their amenability to genetic manipulation. Gene targeting by homologous recombination in hPSCs has proven possible recently using “genome editing” techniques (Lombardo, 2007; Moehle, 2007; Hockemeyer et al., 2009; Zou et al., 2009; Sebastiano et al., 2011; Yusa et al., 2011). However, critical issues remain to be addressed. HPSCs have to be differentiated into a pure and clinical grade population of neural cells of interest regarding the disease. This purification can be achieved by selection of the differentiated cells of interest with fluorescence-activated cell sorting approaches or by limiting/blocking the growth of the undifferentiated hPSCs (by apoptosis or suicide gene induction; Bieberich et al., 2004; Fukuda et al., 2006).

The major challenge regarding PSC-based therapy is the safety of these cells when introduced into patients. In fact, the tumorigenicity of hESCs and hiPSCs is the major hurdle for their application in regenerative medicine (Blum and Benvenisty, 2009; Knoepfler, 2009). Both hPSCs have been shown to form more aggressive tumors than teratoma, the so-called teratocarcinomas (Yang et al., 2008; Blum and Benvenisty, 2009; Werbowetski-Ogilvie et al., 2009; Hovatta et al., 2010). The possible traits of these hPSCs that could induce teratocarcinomas are not completely understood. However, accumulating evidence supports that PSCs show many common similarities with tumor cells and cancer cell lines (Dreesen and Brivanlou, 2007; Knoepfler, 2009) including high proliferation rate, high telomerase activity, and expression of oncogenes (Baker et al., 2007; Hiyama and Hiyama, 2007; Evans and Liu, 2008; Blum and Benvenisty, 2009; Ruggero, 2009; Amps et al., 2011). Several groups reported that the generation of hESCs and hiPSCs were accompanied with somatic coding mutations, copy number variations, and aberrant epigenomic reprogramming (Baker et al., 2007; Gore et al., 2011; Lister et al., 2011). Regarding hESCs, chromosomal aberrations are mostly acquired after culture adaptation over time (Baker et al., 2007). Two types of genomic aberrations can be observed in hESC culture. Transient genomic aberrations eventually appear in culture and disappear after culture passages as they are not advantageous for the hESCs (Hussein et al., 2011). In contrast, stable genomic aberrations that confer growth, self-renewal, and differentiation advantages for hESCs are often selected over time (Baker et al., 2007; Mayshar et al., 2010; Amps et al., 2011). Also, it becomes clearly apparent that genomic stability of hESCs is dependent on culture conditions such as feeder cells, culture medium, cell passaging, freezing, and thawing procedures (Lefort et al., 2009; Olariu et al., 2010). For example, passaging hESCs by “manual cutting and pasting” appears to give more stable cells with a normal karyotype than enzymatic harvesting methods (Buzzard et al., 2004; Mitalipova et al., 2005; Olariu et al., 2010). Among the aberrations observed in hESC lines, gain of chromosomes 12, 17, 20, and X are the most common changes reported (Buzzard et al., 2004; Draper et al., 2004; Maitra et al., 2005; Mitalipova et al., 2005; Spits et al., 2008; Lefort et al., 2009; Hovatta et al., 2010; Mayshar et al., 2010). Recently, the International Stem Cell Initiative analyzed 125 hESC and 11 iPSC lines from 38 laboratories worldwide for genetic changes that occur during culture in which they identified a chromosome 20 minimal amplicon conferring growth advantage (Amps et al., 2011). All these changes are of clinical importance as they have been also described in germ cell tumors and embryonal carcinoma cells (Baker et al., 2007; Blum and Benvenisty, 2009) and could explained the high malignancy of these cells after injection *in vivo*. In line with this, we identified genomic changes acquired in culture that are potentially oncogenic in four hESCs and a teratocarcinoma-like hESC. Among the altered genes, we identified those associated with leukemia translocations and those that promote tumor formation in breast and in urothelial cancers (Hovatta et al., 2010).

Regarding hiPSCs, chromosomal aberrations can originate from the somatic cell before reprogramming (after prolonged time in culture for example), be induced during the reprogramming process and after extended culture of the hiPSCs. Gore et al. investigated the genetic fidelity of 22 hiPSC lines generated by different laboratories using different reprogramming methods. Importantly, coding point mutations were found in all hiPSCs with an average of five protein-coding point mutations. More than 50% of these mutations were also present in the parental fibroblasts while the others were induced during or after the reprogramming process. The majority of these coding point mutations were enriched in genes mutated or involved in cancers (Gore et al., 2011). Moreover, the same chromosomal aberrations described for hESCs have been also reported for hiPSCs (Mayshar et al., 2010; Taapken et al., 2011). In particular, Mayshar and colleagues found that prolonged time in culture is responsible for the duplication of chromosome 12, which is the most common aberration observed in hiPSCs. This adaptation of hiPSCs to culture was associated with the increased expression of critical genes in chromosome 12 including those involved in pluripotency and cell cycle pathways such as *Nanog* and *Growth/differentiation factor 3* (*GDF3*; Mayshar et al., 2010). In line with this, we recently highlighted the crucial role of *Nanog* during reprogramming of somatic cells into hiPSCs with respect to germ cell tumor formation (Grad et al., 2011).

Accumulating evidence suggests that reprogramming of somatic cells into hiPSCs is accompanied with genetic and epigenetic changes (Gore et al., 2011; Lister et al., 2011) that may increase the tumorigenicity of these cells. The first suspects are genes used for reprogramming that are known to be oncogenes such as *klf4* and *c-myc* (Ruggero, 2009). In fact, the reactivation of *c-myc* in iPSC-derived chimeras has been shown to induce tumor formation in mice (Okita et al., 2007; Markoulaki et al., 2009). Reprogramming of somatic cells into iPSCs has been also achieved in the absence of *klf4* and *c-myc* though with a lower efficiency (Huangfu et al., 2008; Nakagawa et al., 2008; Wernig et al., 2008b). However, tumor formation has been described using only *Oct4* as reprogramming factor (Hochedlinger et al., 2005). Another potential risk for tumorigenicity concerns the use of lentiviruses and retroviruses for somatic cell reprogramming. To overcome the potential insertional mutagenesis induced by these methods and the incomplete silencing of reprogramming factor following differentiation (Ramos-Mejia et al., 2010), a number of alternative methods have been developed (see Reprogramming of Somatic Cells into a Pluripotent State). However, the efficiency of these reprogramming methods is very low, in a range of 0.001%. Recently, several groups have developed doxycycline-induced lentiviral vectors that allow their excision by Cre recombinase after cell reprogramming (Kaji et al., 2009; Soldner et al., 2009; Sommer et al., 2010). This method enables the elimination of the transgene expression with a high efficiency of reprogramming.

## Conclusion and Future Perspectives

We believe that hPSC technology provides a promising alternative model to study the pathogenesis of human diseases as it is possible to generate cellular models for most of human diseases. It provides a unique opportunity to generate human cellular models for diseases for which a model is missing (or at least relevant human model). It also limits the use of mouse models in research and drug screening. Moreover, the new experimental finding in generating hiPSCs by reprogramming somatic cells to embryonic stem cell-like (Takahashi et al., 2007; Yu et al., 2007; Park et al., 2008) and to differentiate it into several lineages have opened the possibility to understand the pathogenesis of human diseases. *In vitro* differentiation of such cells may provide unique opportunities for regenerative medicine by generating transplantable cells without immunological rejection. Eventually, it should be possible to treat the defect associated with the disease by pharmacological and gene repair manipulation approaches before transplantation. Finally, these hiPSCs provide an interesting model for pharmacological therapies and for deciphering the molecular targets of therapy response and resistance in humans. Nevertheless, despite those advantages, several issues remain to be solved before their clinical use such as the genomic aberrations and the tumorigenicity of these cells. Therefore, further studies are needed to address whether these cells fulfill their promise in regenerative medicine.

## Conflict of Interest Statement

The authors declare that the research was conducted in the absence of any commercial or financial relationships that could be construed as a potential conflict of interest.

## Abbreviations

AD, Alzheimer’s disease; ALS, amyotrophic lateral sclerosis; AS, Angelman syndrome; CNS, central nervous system; DA, dopaminergic; DS, Down syndrome; EBs, embryoid bodies; ESCs, embryonic stem cells; FD, familial dysautonomia; FDA, U.S. Food and Drug Administration; FRDA, Friedreich’s ataxia; FXD, fragile X syndrome; HD, Huntington’s disease; HPRT, hypoxanthine-guanine phosphoribosyltransferase; iPSCs, induced pluripotent stem cells; MSCs, mesenchymal stem cells; NPCs, neural progenitor cells; NSCs, neural stem cells; PD, Parkinson’s disease; PGD, pre-implantation genetic diagnosis; PSCs, pluripotent stem cells; PWS, Prader–Willi syndrome; RTT, Rett syndrome; SCZD, schizophrenia; SMA, spinal muscular atrophy; SOD, superoxide dismutase; TALENs, transcription activator-like effector nucleases; VLCFA, very long chain acids; X-ALD, X-linked adrenoleukodystrophy-iPSC; ZFNs, zinc finger nucleases.
